# Structural and functional annotation of hypothetical proteins of human adenovirus: prioritizing the novel drug targets

**DOI:** 10.1186/s13104-017-2992-z

**Published:** 2017-12-06

**Authors:** Muhammad Naveed, Sana Tehreem, Muhammad Usman, Zoma Chaudhry, Ghulam Abbas

**Affiliations:** 1 Department of Biotechnology, Faculty of Life Sciences, University of Central Punjab, Lahore, 54000 Pakistan; 2 Department of Biochemistry and Biotechnology, University of Gujrat, Gujrat, 50700 Pakistan

**Keywords:** Human adenovirus, Hypothetical proteins, Function annotation, DNA terminal protein, DNA polymerase, DNA binding protein

## Abstract

**Objective:**

Human adenoviruses are small double stranded DNA viruses that provoke vast array of human diseases. Next generation sequencing techniques increase genomic data of HAdV rapidly, which increase their serotypes. The complete genome sequence of human adenovirus shows that it contains large amount of proteins with unknown cellular or biochemical function, known as hypothetical proteins. Hence, it is indispensable to functionally and structurally annotate these proteins to get better understanding of the novel drug targets. The purpose was the characterization of 38 randomly retrieved hypothetical proteins through determination of their physiochemical properties, subcellular localization, function, structure and ligand binding sites using various sequence and structure based bioinformatics tools.

**Results:**

Function of six hypothetical proteins P03269, P03261, P03263, Q83127, Q1L4D7 and I6LEV1 were predicted confidently and then used further for structure analysis. We found that these proteins may act as DNA terminal protein, DNA polymerase, DNA binding protein, adenovirus E3 region protein CR1 and adenoviral protein L1. Functional and structural annotation leading to detection of binding sites by means of docking analysis can indicate potential target for therapeutics to defeat adenoviral infection.

**Electronic supplementary material:**

The online version of this article (10.1186/s13104-017-2992-z) contains supplementary material, which is available to authorized users.

## Introduction

Human adenoviruses are non-enveloped dsDNA viruses of almost 35 kb in size [1]. HAdV can infect a variety of tissues and cause a wide range of complications like gastroenteritis, hepatitis, myocarditis, keratoconjunctivitis and pneumonia [2, 3]. It is contagion in nature which occurs through direct contact or fomites and virus is also resistant to various physical and chemical agents. Children younger than the age of 5 years and immune compromised persons especially the pediatric patients are most susceptible to these viruses. Worldwide 5–7% respiratory tract infections are ascribed by HAdV in pediatric patients [4] and persons of all ages are susceptible to infections caused by these viruses [5].

Seven known Human adenoviruses species from HAdV-A to HAdV-G are constitute of the genus *Mastadenovirus* in which all the human adenoviruses are categorized and further divided into different strains [6]. Now 67 types of HAdV have been reported [7]. Their number is rapidly increasing due to bioinformatics and genomic advances and availability of whole genome sequences [8, 9].

After an immense effort 50–60% genes have a known function in most of completely sequenced genomes. Number of genes having unknown functions called as hypothetical protein are present in each organism’s genome [10]. To understand the biology and genome of the organisms, it is important to discover the function of hypothetical proteins, despite HAdV has a small size genome but still it has a several hypothetical proteins. So, in order to treat infectious diseases such as those caused by HAdV, functional annotation of these HPs might open avenues for prioritizing novel drug targets [4].

In-silico strategies to annotate the hypothetical proteins are cost effective and fast enough to explore their function. In this study, multiple algorithm based software’s have been used for the prediction of hypothetical protein function that may lead to the identification of novel pharmacological targets for screening, drug discovery and designing for the treatment of HAdV infections [11].

## Main text

### Methods

#### Sequence retrieval

**Fig. 1:**
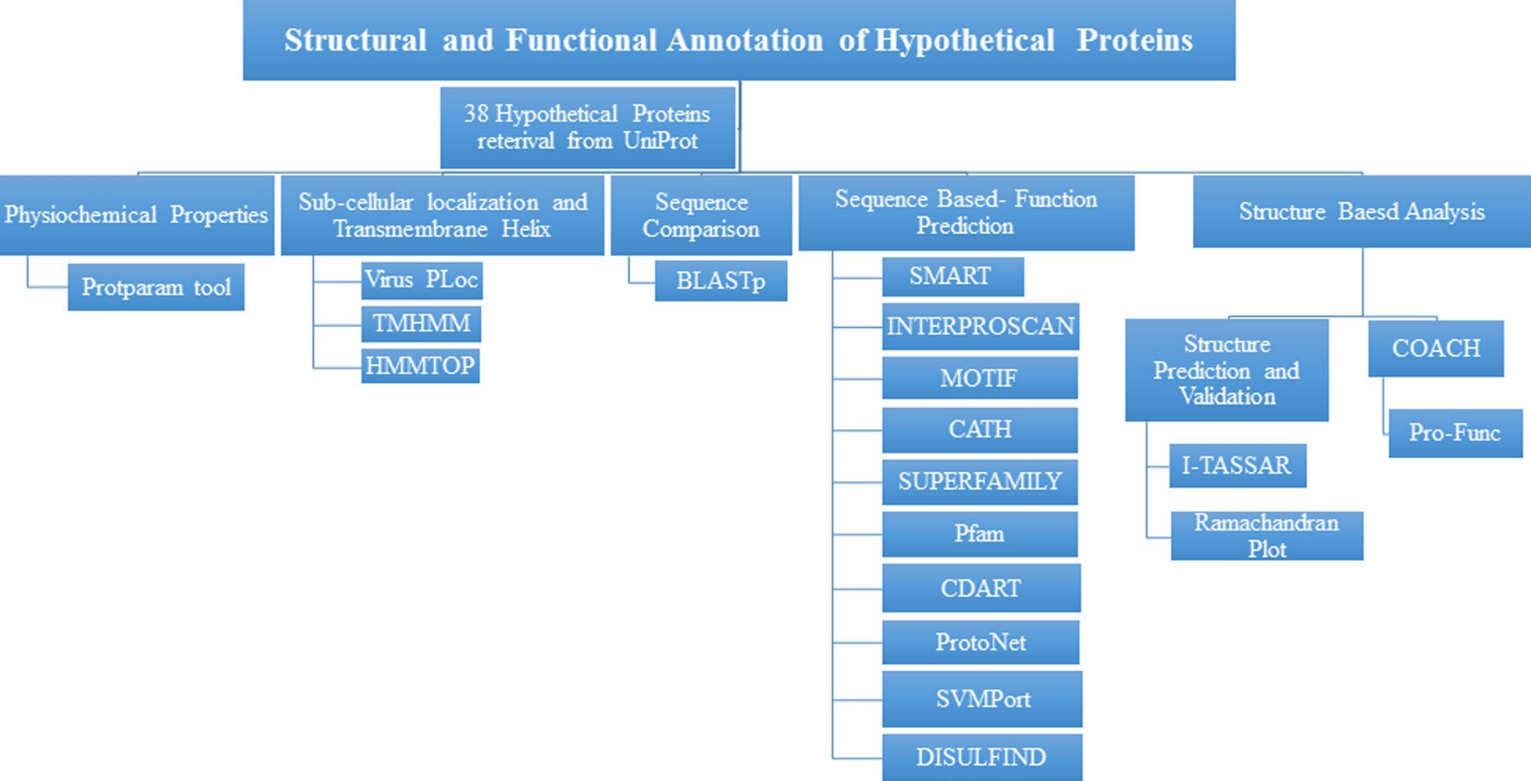
Flowchart that shows all of the tools used for functional and structural annotation of hypothetical proteins

Proteins having unknown function of Human adenovirus were taken from UniProt [12, 13]. Random selection of 38 hypothetical proteins belonging to eight different types of HAdV was carried out (Additional file 1: Table S1). The sequence analysis was done by taking FASTA sequence of these proteins along with their UniProt ID. For characterization purposes, number of software based on different algorithms were used as shown in Fig. 1.

#### Physicochemical characterization

Analysis of physiochemical properties of all HPs was done by online server ExPASy’s Protparam tool [14]. This server executes theoretical evaluation of physiochemical properties like isoelectric point, molecular weight, aliphatic index, grand average of hydropathicity (GRAVY) and instability index [15].

#### Sub-cellular localization

To predict the cellular function of a protein it is important to get information about its sub-cellular localization i.e. a protein can be present in outer membrane, inner membrane, periplasm, extracellular space or in cytoplasm [16]. Sub-cellular localization of viral proteins were predicted using Virus-PLoc [17] online server tool [18], TMHMM [19, 20] and HMMTOP [21, 22].

#### Sequence analogy

Most basic step in the function prediction of a protein is looking for its structural homologs in different available genomics and proteomics based databases. Popular bioinformatics tool BLASTp was used for this purposes [23, 24].

#### Function and disulfide bridges prediction

For precise function annotation, various tools like SVMport, ProtNet [25, 26], Pfam, Motif [27, 28], CDART [24, 29], CATH [30, 31], SMART [32, 33], Superfamily [34, 35] and InterProscan [27, 36] were used that classified all 38 proteins of HAdV into families and subfamilies on the basis of their sequence, structure and function [16, 37]. DISULFIND [38] server was used to evaluate occurrence of disulfide bonds between cysteine residues [39].

#### Structure prediction and validation

For prediction and validation of three dimensional I-TASSAR (Iterative Threading Assembly Refinement) [40, 41] Ramachandran Plot were used [42, 43].

#### Structure analysis

Functions of proteins based on structural analysis are considered more acceptable as compared to sequence based function annotation, because homologous proteins show more conserved structures in evolution than sequences [44]. For this purpose, we have used ProFunc [27] and COACH [40, 45].

### Results

Random selection of 38 hypothetical proteins belonging to eight different types of HAdV was carried out from UniProt (Additional file 1: Table S1). The amino acid length of 38 randomly selected proteins of eight different types of Human Adenovirus ranges from 1198 amino acids for longest protein to 81 amino acids for shortest protein (Additional file 1: Table S1). Protparam tool has been used for the prediction of physiochemical properties of all hypothetical proteins (Additional file 2: Table S2). Subcellular localization and transmembrane helix prediction software predicated most of the HPs to be localized in the host cytoplasm and a few in-host cell membrane and nucleus (Additional file 3: Table S3). Multiple softwares were used for the function prediction of 38 hypothetical proteins (Additional file 4: Table S4, Additional file 5: Table S5). Out of 38 proteins, 6 HP’s whose function was confidently predicted by ≥ 6 software’s were confidently selected (Table 1). Confidently function predicted HPs were further used for structure prediction, structure analysis and disulphide bridges prediction. The detailed results of structure prediction and analysis are shown in Additional file 6: Table S6 and Additional file 7: Table S7. DISULFIND was unable to find disulphide bonds in any of the HP’s and characterized them as thermally unstable proteins.

**Table 1:** Proteins whose function is predicted confidently along with their corresponding genomes and subcellular location

Sr no	Uniprot ID	HAdV	Function	Confidence level	Virus-PLoc
01	P03269	HAdV-2	DNA terminal protein	7/9	Host nucleus
02	P03261	HAdV-2	DNA polymerase	8/9	Host nucleus
03	P03263	HAdV-2	DNA binding protein	6/9	Host cytoplasm
04	Q83127	HAdV-7	Adenovirus E3 region protein CR1	6/9	Host cell membrane
05	Q1L4D7	*Human mastadenovirus B*	Adenoviral protein L1	7/9	Host cytoplasm and nucleus
06	I6LEV1	*Human mastadenovirus B*	Adenoviral protein L1	6/9	Host cytoplasm and nucleus

### Discussion

In this study, we carried out structural and functional annotation of 38 HPs of human adenovirus that is responsible for variety of clinical diseases. Physiochemical properties prediction showed that Isoelectric point [46] of HPs ranges from 4.1 to 12.43. Isoelectric point is pH at which the net charge on the protein is zero and at this pH the protein become less soluble, compact and stable that leads to crystallization of protein. So, the purification and crystallization of protein can be carried out by developing a buffer system with the help of computed pI  [47, 48] (Additional file 2: Table S2).

The extinction coefficient of the HPs computed by Protparam tool ranges from 1490.0 to 179,580.0 M^−1^ cm^−1^ at 280 nm. This computed extinction coefficient can be helpful for quantitatively studying protein–ligand and protein–protein interaction. It is forecasted that if the instability index is less than 40 then a protein will be stable and if greater than 40 then it will be unstable. The instability index of 38 hypothetical proteins ranges from 20.1 to 106.56 and due to this only nine proteins are stable and rest is unstable. The GRAVY index of all proteins ranges from − 0.908 to 0.166 and out of 38 HPs, 32 HPs have negative GRAVY index which indicate that these proteins are non-polar in nature [49].

The detailed information about the functional and structural annotation for six hypothetical proteins is as follow:

#### P03269

P03269 is predicted as an adenoviral DNA terminal protein that performs function in the initiation of the viral DNA replication [50]. This protein is covalently bound to the viral DNA and acts as a primer for viral genomic replication by DNA strand displacement [51]. Seven software confidently predicted the function of this protein and Virus-PLoc server also confirmed its function by predicting its location in host nucleus. Predicted three-dimensional structure highest C-score − 2.25 (Additional file 8: Figure S1) was selected and structure verification through RAMACHANDRAN PLOT showed 76.9% residues are in most favored region and 18.8% residue are in additional allowed region (Additional file 9: Figure S2). For pharmaceutical and docking analysis, COACH has been used, out of many ligand binding sites, best ligand binding sites with maximum C-score were selected that can be used for further molecular docking analysis (Additional file 6: Table S6). Further structure based function analysis predicted adenoviral DNA terminal protein motif in HP P03269 and Ala159-Arg161, Gly558-Gly560, Leu406-Glu408, Gln241-Ala243 and Pro275-Arg277 structure motifs are also predicted to be conserved in this HP that may have a similar function (Additional file 7: Table S7). Gene Ontology analysis shows that HP P03269 may have role in the biological process of DNA replication, cellular process, cellular metabolic process, cellular biosynthetic process and biochemical function as DNA binding and nucleic acid binding.

#### P03261

P03261 belongs to *Human adenovirus C serotype 2* and predicted to contain DNA polymerase type-B family catalytic domain and sub-cellularly localized in host nucleus. DNA-directed DNA polymerases has both exonucleases and polymerase activity and play role in the process of recombination, repair and DNA replication [52]. Out of five 3D models predicted by I-TASSAR, structure with highest C-score (− 0.20) was selected (Additional file 10: Figure S3) and structure verification shows that 67.1% residues are in favored region and 25.7% residues are in additional allowed regions of RC-plot (Additional file 11: Figure S4). ProFunc server has predicted DNA polymerase family B signature.

Gene ontology analysis showed that this HP may play its role in DNA replication and cellular process and biochemically function in nucleotide binding, nucleic acid binding and DNA-directed DNA-polymerase activity. DNA polymerase type B, organellar and viral and DNA-directed DNA-polymerase family B signature motifs in the HP these results have further validated the results of sequence based function prediction. Five other structure motifs were also identified as Leu323-Asp326, His955-Leu957, Ser926-Pro928, Leu645-Pro647 and Lys850-Asn853 (Additional file 7: Table S7).

#### P03263

P03263 is predicted as an adenoviral protein L1 52/55-kDa and that perform multiple functions in DNA packaging by facilitating stable interactions between empty capsid and viral DNA through its expression both in the early and late stages of infection cycle [46] (Additional file 12: Figure S5).

Model with highest C-score − 3.74 was selected and structure validation shows that 69.6% residues are in favored regions and 23.2% resides are present in the additional allowed regions of RC-plot (Additional file 13: Figure S6). Functional analysis server has verified the results of sequence based function prediction by predicting adenoviral protein L1 52/55-kDa motif in HP P03263 along with three conserved structure motifs Ala105-Ala107, Glu7-Asp9 and Asp4-Glu6 (Additional file 7: Table S7). According to gene ontology results HP P03263, HPQ83127, HP I6LEV1 are involved in the biological process of virion assembly, anatomical structure formation, anatomical structure formation involved in morphogenesis and cellular component assembly involved in morphogenesis.

#### Q83127

Q83127 is annotated as Adeno E3 region protein CR1 that is responsible for controlling the viral interactions with host [53]. The virus-PLoc also confirmed that this protein is a transmembrane and HMMTOP predicts 2 helices in a membrane. Three-dimensional structure with highest C-score − 4.78 (Additional file 14: Figure S7) was selected and structure verification using SAVES shows that 42.9% residues are in favored region and 44.1% residues are in additional allowed regions (Additional file 15: Figure S8). HP contains Adenovirus E3 region protein CR2 and Adenovirus E3 region protein CR1 motifs along with one conserved structural motif Gln171-Pro173 (Additional file 7: Table S7).

#### Q1L4D7

Q1L4D7 is predicted as adenoviral protein L1 and confidence level for this HP is seven out of nine respectively. This protein expresses in both early and late stage of viral life cycle and plays multiple roles in DNA packaging [46]. We have modeled its three-dimensional structure and out of five models with C-score − 4.53 (Additional file 16: Figure S9) was selected. RC-plot shows that 34.7% residues are in favored region and 46.0% are present in the additional allowed regions (Additional file 17: Figure S10) and contains two structure motifs Glu65-Ala67 and Val115-Gly117 (Additional file 7: Table S7).

#### I6LEV1

I6LEV1 is also predicted as adenoviral protein L1 like HP Q1L4D7. Structure verification of model with C-score − 4.56 (Additional file 18: Figure S11) shows that 36.0% residues are in favored regions and 43.2% residues are in additional allowed regions of RC-plot (Additional file 19: Figure S12). Sequence based function prediction verified by structural analysis and predicted three structural motifs Leu22-Leu24, Val98-Glu100 and Arg126-His128 (Additional file 7: Table S7).

To summarize, this study helped to search functionality in the hypothetical proteins of human adenovirus whose exact role in the infectious cycle was still unknown. Finally, we may emphasize that quantitative computational analysis that is carried out in the present study, may help us in better understanding of the biology of adenovirus as a whole and identify potential therapeutic leads to molecular level and may facilitate better understanding of the human biology.

### Limitations

As our study is based on less sample size, increase sample size can provide more information about the function of HPs proteins and for identifying novel drug targets and this study is totally based on in silico analysis but through side by side wet lab analysis these proteins can be used for drug targeting analysis on experimental basis.

## Additional files

**Additional file 1: Table S1.** This table reports list of 38 hypothetical proteins of human adenovirus along with their UniProt ID, corresponding genome and protein length.

**Additional file 2: Table S2.** This table presents list of predicted physiochemical properties of 38 human adenovirus hypothetical proteins.

**Additional file 3: Table S3.** This table reports list of predicted sub-cellular localization of the 38 HPs from human adeno viruses (HADVs).

**Additional file 4: Table S4.** This table details list of annotated function of 38 human adenovirus using BLASTp, SMART, INTERPROSCAN and MOTIF.

**Additional file 5: Table S5.** This table presents list of functionally annotated domain and motifs of HPs 38 s from human adenovirus by CATH, SUPERFAMILY, Pfam, CDART, ProtNet and SVMprot.

**Additional file 6: Table S6.** This table presents ligand binding sites prediction of hypothetical proteins of human adenovirus.

**Additional file 7: Table S7.** Sequence and structure motif prediction by pro-func.

**Additional file 8: Figure S1.** 3D structure of hypothetical protein P03269 predicted from I-TASSER.

**Additional file 9: Figure S2.** Evaluation of 3D structure of hypothetical protein P03269 through Ramachandran Plot.

**Additional file 10: Figure S3.** 3D structure of hypothetical protein P03261 predicted from I-TASSER.

**Additional file 11: Figure S4.** Evaluation of 3D structure of Hypothetical Protein P03261 through Ramachandran Plot

**Additional file 12: Figure S5.** 3D structure of hypothetical protein P03263 predicted from I-TASSER.

**Additional file 13: Figure S6.** Evaluation of 3D structure of hypothetical protein P03263 through Ramachandran Plot.

**Additional file 14: Figure S7.** 3D structure of hypothetical protein Q83127 predicted from I-TASSER.

**Additional file 15: Figure S8.** Evaluation of 3D structure of hypothetical protein Q83127 through Ramachandran Plot.

**Additional file 16: Figure S9.** 3D structure of hypothetical protein Q1L4D7 predicted from I-TASSER.

**Additional file 17: Figure S10.** Evaluation of 3D structure of hypothetical protein Q1L4D7 through Ramachandran Plot.

**Additional file 18: Figure S11.** 3D structure of hypothetical protein 16LEV1 predicted from I-TASSER.

**Additional file 19: Figure S12.** Evaluation of 3D structure of hypothetical protein I6LEV1 through Ramachandran Plot.

## Abbreviations

HAdV: human adenoviruses
HP: hypothetical protein
GRAVY: grand average of hydropathicity
I-TASSAR: iterative threading assembly refinement
