# Supplementary material for: Structural and functional annotation of hypothetical proteins of human adenovirus: prioritizing the novel drug targets
Source: BMC Res Notes. 2017 Dec 6;10:706. doi: 10.1186/s13104-017-2992-z (PMC5719520; doi:10.1186/s13104-017-2992-z)
Supplement: Supplementary file 1 — Additional file 1: Table S1. This table reports list of 38 hypothetical proteins of human adenovirus along with their UniProt ID, corresponding genome and protein length. [file 13104_2017_2992_MOESM1_ESM.docx]

| **Table S1: List of 38 Hypothetical Proteins of Human Adenovirus along with their Uniprot ID, corresponding genome and protein length** | | | |
| --- | --- | --- | --- |
| Sr No | **UniProt ID** | **HAdV** | **Protein Length** |
| 01 | P03269 | HAdV-2 | 671 |
| 02 | P03261 | HAdV-2 | 1198 |
| 03 | P03263 | HAdV-2 | 145 |
| 04 | Q83127 | HAdV-7 | 179 |
| 05 | P03287 | HAdV-2 | 106 |
| 06 | P03289 | HAdV-2 | 112 |
| 07 | P03294 | HAdV-2 | 121 |
| 08 | P03292 | HAdV-2 | 168 |
| 09 | P03291 | HAdV-2 | 215 |
| 10 | P03293 | HAdV-2 | 137 |
| 11 | Q1L4D7 | Human mastadenovirus B | 146 |
| 12 | I6LEV1 | Human mastadenovirus B | 146 |
| 13 | E1ARQ3 | Human mastadenovirus C | 121 |
| 14 | Q4JEP5 | HAdV-7 | 81 |
| 15 | Q5EY75 | HAdV-7 | 114 |
| 16 | Q2KS67 | HAdV-7 | 173 |
| 17 | E1U5M6 | HAdV-6 | 112 |
| 18 | E1U5N2 | HAdV-6 | 176 |
| 19 | A0A0B4SHT8 | Human adenovirus 21 | 104 |
| 20 | A0A0B4SJJ5 | Human adenovirus 21 | 114 |
| 21 | A0A0B4SI61 | Human adenovirus 21 | 173 |
| 22 | A0A0B4SHQ0 | Human adenovirus 21 | 114 |
| 23 | Q3ZKV3 | Human adenovirus 50 | 104 |
| 24 | Q5EY73 | HAdV-7 | 133 |
| 25 | Q2KS66 | HAdV-7 | 95 |
| 26 | I1V173 | HAdV-7 | 91 |
| 27 | Q2KS62 | HAdV-7 | 133 |
| 28 | A0A0B4SIA5 | Human adenovirus 21 | 91 |
| 29 | A0A0B4SGV2 | Human adenovirus 21 | 133 |
| 30 | A0A0B4SIU9 | Human adenovirus 21 | 133 |
| 31 | Q2KS78 | Human adenovirus 21 | 95 |
| 32 | Q2KSC0 | Human adenovirus 21 | 91 |
| 33 | A0A0B4SH32 | Human adenovirus 21 | 106 |
| 34 | Q3ZKV7 | Human adenovirus 50 | 173 |
| 35 | Q3ZKV4 | Human adenovirus 50 | 133 |
| 36 | Q3ZKV2 | Human adenovirus 50 | 91 |
| 37 | A6MLW9 | Human mastadenovirus E | 106 |
| 38 | E1U5M8 | HAdV-6 | 215 |
