# Supplementary material for: Structural and functional annotation of hypothetical proteins of human adenovirus: prioritizing the novel drug targets
Source: BMC Res Notes. 2017 Dec 6;10:706. doi: 10.1186/s13104-017-2992-z (PMC5719520; doi:10.1186/s13104-017-2992-z)
Supplement: Supplementary file 2 — Additional file 2: Table S2. This table presents list of predicted physiochemical properties of 38 human adenovirus hypothetical proteins. [file 13104_2017_2992_MOESM2_ESM.docx]

| **Table S2: List of Predicted Physiochemical Properties of 38 Human Adenovirus Hypothetical Proteins** | | | | | | | | |
| --- | --- | --- | --- | --- | --- | --- | --- | --- |
| **S.No** | **Uniprot Id** | **Molecular weight Mw(Da)** | **Theoretical PI** | **Extinction coefficient**  **(M-1 cm-1)** | **Instability Index** | | **Aliphatic index** | **Grand average of hydropathicity (GRAVY)** |
|  |  |  |  |  |  |  |  |  |
|  |  |  |  |  |  |  |  |  |
|  |  |  |  |  | **Computed** | **Classification** |  |  |
| 1 | P03269 | 76542.7 | 5.93 | 75665.0 | 62.94 | unstable | 80.25 | -0.442 |
| 2 | P03261 | 135669.9 | 7.17 | 179580.0 | 47.37 | unstable | 75.84 | -0.483 |
| 3 | P03263 | 16102.4 | 5.95 | 26595.0 | 45.28 | unstable | 102.83 | 0.037 |
| 4 | P03287 | 11668.2 | 12.18 | 7240.0 | 95.64 | unstable | 50.57 | -0.76 |
| 5 | P03289 | 12811 | 12.43 | 8480.0 | 63.45 | unstable | 70 | -0.855 |
| 6 | P03294 | 12755.7 | 11.38 | 12615.0 | 67.77 | unstable | 74.46 | -0.417 |
| 7 | P03292 | 17703 | 12.25 | 16625.0 | 106.56 | unstable | 54.58 | -0.6 |
| 8 | P03291 | 22787.7 | 12.03 | 25355.0 | 98.63 | unstable | 36.09 | -0.905 |
| 9 | P03293 | 14356.1 | 10.04 | 23615.0 | 41.03 | unstable | 66.93 | -0.331 |
| 10 | E1U5M6 | 12777 | 12.43 | 8480.0 | 63.45 | unstable | 73.48 | -0.846 |
| 11 | E1U5N2 | 18702.5 | 11.6 | 14230.0 | 58.09 | unstable | 61.25 | -0.539 |
| 12 | E1U5M8 | 22815.7 | 12.06 | 25355.0 | 100.32 | unstable | 36.09 | -0.908 |
| 13 | Q83127 | 20020.6 | 9.3 | 20650.0 | 38.5 | unstable | 106.76 | 0.27 |
| 14 | Q4JEP5 | 8779.8 | 11.72 | 3150.0 | 59.2 | unstable | 31.6 | -1.044 |
| 15 | Q5EY75 | 12607 | 11.61 | 4595.0 | 65.51 | unstable | 31.84 | -1.28 |
| 16 | Q2KS67 | 19013.9 | 11.49 | 45740.0 | 68.47 | unstable | 58.09 | -0.463 |
| 17 | Q5EY73 | 14556.4 | 4.93 | 1490.0 | 20.83 | stable | 113.61 | -0.162 |
| 18 | Q2KS66 | 10357.5 | 4.19 | 1490.0 | 62.22 | unstable | 110.63 | -0.06 |
| 19 | I1V173 | 9693 | 6.19 | 3105.0 | 20.1 | stable | 88.79 | 0.151 |
| 20 | Q2KS62 | 14557.5 | 5.01 | 3105.0 | 27.07 | stable | 114.36 | -0.071 |
| 21 | Q1L4D7 | 16346.5 | 4.97 | 24200.0 | 49.28 | unstable | 91.44 | -0.053 |
| 22 | I6LEV1 | 16404.6 | 4.86 | 24200.0 | 50.31 | unstable | 91.44 | -0.074 |
| 23 | E1ARQ3 | 12739.7 | 11.38 | 12615.0 | 67.15 | unstable | 71.24 | -0.462 |
| 24 | A6MLW9 | 11494.3 | 12.01 | 7115.0 | 73.92 | unstable | 61.6 | -0.33 |
| 25 | A0A0B4SHT8 | 11513.5 | 9.99 | 23740.0 | 58.73 | unstable | 62.12 | -0.686 |
| 26 | A0A0B4SJJ5 | 12578.9 | 11.61 | 4595.0 | 65.51 | unstable | 30.18 | -1.301 |
| 27 | A0A0B4SI61 | 18939.9 | 11.64 | 40240.0 | 67.98 | unstable | 60.35 | -0.438 |
| 28 | A0A0B4SHQ0 | 12605 | 11.61 | 4595.0 | 63.83 | unstable | 33.6 | -1.261 |
| 29 | Q2KS78 | 10394.6 | 4.1 | 1490.0 | 57.37 | unstable | 110.63 | -0.025 |
| 30 | Q2KSC0 | 9745 | 5.57 | 1615.0 | 32.37 | stable | 88.79 | 0.166 |
| 31 | A0A0B4SIA5 | 9744 | 5.75 | 1615.0 | 28.61 | stable | 88.79 | 0.166 |
| 32 | A0A0B4SGV2 | 14546.4 | 4.93 | 1490.0 | 21.8 | stable | 114.36 | -0.111 |
| 33 | A0A0B4SIU9 | 14545.4 | 5.02 | 1490.0 | 22.73 | stable | 114.36 | -0.111 |
| 34 | A0A0B4SH32 | 11433.2 | 11.92 | 12490.0 | 57.4 | unstable | 72.64 | -0.101 |
| 35 | Q3ZKV3 | 11497.5 | 9.99 | 23740.0 | 56.87 | unstable | 63.08 | -0.661 |
| 36 | Q3ZKV7 | 18884.6 | 11.49 | 40240.0 | 66.6 | unstable | 58.09 | -0.447 |
| 37 | Q3ZKV4 | 14624.5 | 5.13 | 1490.0 | 21.56 | stable | 112.86 | -0.153 |
| 38 | Q3ZKV2 | 9745 | 5.57 | 1615.0 | 32.37 | stable | 88.79 | 0.166 |
