# Supplementary material for: Structural and functional annotation of hypothetical proteins of human adenovirus: prioritizing the novel drug targets
Source: BMC Res Notes. 2017 Dec 6;10:706. doi: 10.1186/s13104-017-2992-z (PMC5719520; doi:10.1186/s13104-017-2992-z)
Supplement: Supplementary file 3 — Additional file 3: Table S3. This table reports list of predicted sub-cellular localization of the 38 HPs from human adeno viruses (HADVs). [file 13104_2017_2992_MOESM3_ESM.docx]

| **Table S3: List of Predicted Sub-Cellular Localization of the 38 HPs from Human Adeno Viruses (HADVs)** | | | | |
| --- | --- | --- | --- | --- |
| **Sr No** | **Uniprot id** | **Virus-ploc** | **HMMTOP** | **TMHMM** |
| 01 | P03269 | Host nucleus | Not present | Not present |
| 02 | P03261 | Host nucleus | 1 TMHs | Not present |
| 03 | P03263 | Host Cytoplasm | 2 TMHs | 2 TMHs |
| 04 | P03287 | Host Cytoplasm | Not present | Not present |
| 05 | P03289 | Host Cytoplasm | Not present | Not present |
| 06 | P03294 | Host Cytoplasm | Not present | Not present |
| 07 | P03292 | Host Cytoplasm | Not present | Not present |
| 08 | P03291 | Host Cytoplasm | Not present | Not present |
| 09 | P03293 | Host Cytoplasm | Not present | Not present |
| 10 | E1U5M6 | Host Cytoplasm | Not present | Not present |
| 11 | E1U5N2 | Host cytoplasm | not present | Not present |
| 12 | E1U5M8 | Host cytoplasm | Not present | Not present |
| 13 | Q83127 | Host cell membrane | 2 TMHs | Not present |
| 14 | Q4JEP5 | Host Cytoplasm | Not present | Not present |
| 15 | Q5EY75 | Host Cytoplasm | Not present | Not present |
| 16 | Q2KS67 | Host cell membrane | Not present | Not present |
| 17 | Q5EY73 | Host Cytoplasm | Not present | Not present |
| 18 | Q2KS66 | Host Cytoplasm | Not present | Not present |
| 19 | I1V173 | Host Cytoplasm | 1 TMHs | Not present |
| 20 | Q2KS62 | Host cytoplasm | Not present | Not present |
| 21 | Q1L4D7 | Host Cytoplasm  Host nucleus | 1 TMHs | 1 TMHs |
| 22 | I6LEV1 | Host Cytoplasm  Host nucleus | Not present | 1 TMHs |
| 23 | E1ARQ3 | Host Cytoplasm | Not present | Not present |
| 24 | A6MLW9 | Host Cytoplasm | Not present | Not present |
| 25 | A0A0B4SHT8 | Host cell membrane  Host Cytoplasm | Not present | Not present |
| 26 | A0A0B4SJJ5 | Host Cytoplasm | Not present | Not present |
| 27 | A0A0B4SI61 | Host cell membrane | Not present | Not present |
| 28 | A0A0B4SHQ0 | Host Cytoplasm | Not present | Not present |
| 29 | Q2KS78 | Host Cytoplasm | Not present | Not present |
| 30 | Q2KSC0 | Host Cytoplasm | Not present | Not present |
| 31 | A0A0B4SIA5 | Host Cytoplasm | 1 TMHS | Not present |
| 32 | A0A0B4SGV2 | Host Cytoplasm | Not present | Not present |
| 33 | A0A0B4SIU9 | Host Cytoplasm | Not present | Not present |
| 34 | A0A0B4SH32 | Host Cytoplasm | Not present | Not present |
| 35 | Q3ZKV3 | Host cell membrane  Host Cytoplasm | Not present | Not present |
| 36 | Q3ZKV7 | Host cell membrane | Not present | Not present |
| 37 | Q3ZKV4 | Host Cytoplasm | Not present | Not present |
| 38 | Q3ZKV2 | Host Cytoplasm | Not present | Not present |
