# Supplementary material for: Structural and functional annotation of hypothetical proteins of human adenovirus: prioritizing the novel drug targets
Source: BMC Res Notes. 2017 Dec 6;10:706. doi: 10.1186/s13104-017-2992-z (PMC5719520; doi:10.1186/s13104-017-2992-z)
Supplement: Supplementary file 4 — Additional file 4: Table S4. This table details list of annotated function of 38 human adenovirus using BLASTp, SMART, INTERPROSCAN and MOTIF. [file 13104_2017_2992_MOESM4_ESM.docx]

| **Table S4: List of Annotated Function of 38 Human Adenovirus Using BLASTp, SMART, INTERPROSCAN and MOTIF** | | | | | |
| --- | --- | --- | --- | --- | --- |
| **S.NO** | **UNIPROT ID** | **Major BLAST hit** | **SMART** | **INTERPROSCAN** | **MOTIF** |
|  | P03269 | DNA terminal protein precursor | Adeno_terminal | Adenoviral preterminal protein | Adenoviral DNA terminal protein |
|  | P03261 | DNA polymerase function in DNA replication | POLBc | DNA-directed DNA polymerase, family B | 1. DNA polymerase type B  2. DNA polymerase family B  3. 3'-5' exonuclease |
| **3** | P03263 | DNA binding protein/I leader protein | Adeno_52K | Adenoviral 52/55kDa protein | 1.Adenoviral protein L1 52/55-kDa  2.OpgC protein |
|  | P03287 | No result | No result | Unintegrated | No result |
|  | P03289 | Uncharacterized protein F-112 | No result | No result | No result |
|  | P03294 | Uncharacterized F-121 | No result | Unintegrated | No result |
|  | P03292 | No result | No result | Unintegrated | No result |
|  | P03291 | DNA terminal Protein | No result | No result | No result |
|  | P03293 | No result | No result | No result | No result |
|  | E1U5M6 | No results | No result | No result | No result |
|  | E1U5N2 | No result | No result | Unintegrated | No result |
|  | E1U5M8 | DNA terminal protein | No result | No result | No result |
|  | Q83127 | Membrane glycoprotein E3 CR1-beta | Adeno_E3_CR1  Adeno_E3_CR2 | 1. Adenovirus E3 region protein CR1  2. Adenovirus E3 region protein CR2  Unintegrated | 1.Adenovirus E3 region protein CR1  2.TMEM154 protein family  3.Tweety  4. Myo-inositol-1-phosphate synthase  5. C-terminal domain of neuropilin glycoprotein  6. IncA protein  7. Orthoreovirus membrane fusion protein p10 |
|  | Q4JEP5 | DNA terminal protein | No result | No result | No result |
|  | Q5EY75 | No result | No result | No result | No result |
|  | Q2KS67 | No result | No result | No result | No result |
|  | Q5EY73 | No result | No result | No result | No result |
|  | Q2KS66 | No result | No result | No result | No result |
|  | I1V173 | No result | No result | No result | No result |
|  | Q2KS62 | No result | No result | No result | No result |
|  | Q1L4D7 | No result | Adeno_52K | Adenoviral 52/55kDa protein  Unintregrated | Adenoviral protein L1 52/55-kDa |
|  | I6LEV1 | DNA binding Agnoprotein | Adeno_52K | Adenoviral 52/55kDa protein  unintegrated | Adenoviral protein L1 52/55-kDa |
|  | E1ARQ3 | No result | No result | Unintegrated | No result |
|  | A6MLW9 | No result | No result | Unintegrated | Glycerol-3-phosphate acyltransferase |
|  | A0A0B4SHT8 | No result | No result | No result | No result |
|  | A0A0B4SJJ5 | DNA terminal protein | No result | No result | No result |
|  | A0A0B4SI61 | No result | No result | No result | No result |
|  | A0A0B4SHQ0 | DNA terminal protein | No result | No result | No result |
|  | Q2KS78 | No result | No result | No result | No result |
|  | Q2KSC0 | No result | No result | No result | Type-F conjugative transfer system protein (TrbI_Ftype) |
|  | A0A0B4SIA5 | No result | No result | No result | No result |
| **32.** | A0A0B4SGV2 | No result | No result | No result | No result |
| **33.** | A0A0B4SIU9 | No result | No result | No result | No result |
| **34.** | A0A0B4SH32 | No result | No result | Unintegrated | Glycerol-3-phosphate acyltransferase |
| **35.** | Q3ZKV3 | No result | No result | No result | No |
| **36.** | Q3ZKV7 | No result | No result | No result | No |
| **37.** | Q3ZKV4 | No result | No result | No result | No |
| **38.** | Q3ZKV2 | No result | No result | No result | Type-F conjugative transfer system protein (TrbI_Ftype) |
