# Supplementary material for: Structural and functional annotation of hypothetical proteins of human adenovirus: prioritizing the novel drug targets
Source: BMC Res Notes. 2017 Dec 6;10:706. doi: 10.1186/s13104-017-2992-z (PMC5719520; doi:10.1186/s13104-017-2992-z)
Supplement: Supplementary file 5 — Additional file 5: Table S5. This table presents list of functionally annotated domain and motifs of HPs 38 s from human adenovirus by CATH, SUPERFAMILY, Pfam, CDART, ProtNet and SVMprot. [file 13104_2017_2992_MOESM5_ESM.docx]

| **Table S5: List of Functionally Annotated Domain and Motifs of HPs 38 s from Human Adenovirus by CATH,**  **SUPERFAMILY, Pfam, CDART, Protonet and SVMprot** | | | | | | | | |
| --- | --- | --- | --- | --- | --- | --- | --- | --- |
| **S.NO** | **UNIPROT ID** | **CATH** | **SUPER FAMILY** | **Pfam**  **(family/**  **Domain)** | **CDART** | **Protonet**  **Cluster**  **(cluster name)** | **SVMProt (family)** | |
|  |  |  |  |  |  |  | **Molecular Function** | **Biological Process** |
| 1 | P03269 | No result | No | Adeno_terminal | Adenoviral DNA terminal protein | Cluster A3384322  DNA terminal protein | 1.All DNA binding  2.Zinc binding | DNA replication |
| 2 | P03261 | DNA polymerase | \|  \| [Ribonuclease H-like](http://supfam.org/SUPERFAMILY/cgi-bin/scop.cgi?sunid=53098) \| \| --- \| --- \| | DNA_pol_B_2 | DNA polymerase | Cluster A4145585 DNA directed DNA polymerase family B | 1. DNA directed polymerase   2. Zinc binding | DNA replication |
| 3 | P03263 | No result | No | Adeno_52K | Mediate association between empty and capsid and DNA (leader protein having multiple roles in whole life cycle) | Cluster A3915575 Mast adenovirus  DNA Binding | Metal Binding | Transmembrane  protein |
| 4 | P03287 | No result | No | No result | No result | Cluster A4013018  Adenoviridae early protein | 1. Mg binding  2. mRNA binding protein | Not found |
| 5 | P03289 | No results | No | No result | No result | Cluster A421689 protein of unknown function | 1. mRNA binding protein  2. All DNA binding Protein | 1. DNA replication  2.Neuropeptide |
| 6 | P03294 | No result | No | No result | No result | Cluster A3539386 uncharacterized protein F-112 | 1.Metal binding  2.All DNA binding | 1. DNA replication  2. DNA repair  3.ATP binding cascade  4. (ABC)family broadly defined function |
| 7 | P03292 | No result | No | No result | No result | Cluster A373955 uncharacterized protein-168 | 1. Metal binding DNA binding  Isomerase  2. Intramolecular reductase  P=58.6 | 1. DNA repair  2. Lipid synthesis |
| 8 | P03291 | No result | No | No result | No result | Cluster A4075149 early protein uncharacterized protein F-215 | 1.All DNA binding  2. RNA binding | 1. DNA Repair |
| 9 | P03293 | No result | No | No result | No result | Cluster A725929 uncharacterized protein b-137 | 1. Zinc Binding | Chlorophyll biosynthesis |
| 10 | E1U5M6 | No result | No | No result | No result | Cluster A1677775 hypothetical protein B-137 P-80985 | 1. Type II secretory pathway family  2. RNA binding | Not found |
| 11 | E1U5N2 | No result | No | No result | No result | Cluster A725929 uncharacterized protein b-137 | 1. Zinc binding  2. DNA binding 3. Transferase | Outer membrane |
| 12 | E1U5M8 | No result | No | No result | No result | Cluster A4075149 early protein | 1. Metal binding 2. RNA binding | Not found |
| 13 | Q83127 | Receptor protein kinase | Murd-like peptide ligases, catalytic domain | Adeno_E3_CR1  Adeno_E3_CR2 | Control interaction with host (function unknown) | Cluster 3999666 Adenoviral E3 region protein | All lipid binding protein | Transmembrane |
| 14 | Q4JEP5 | No result | No | No result | No result | Cluster 4075149 uncharacterized protein F-115 | 1. Channels pore forming toxins  2. Hydrolases  3. mRNA binding | DNA replication |
| 15 | Q5EY75 | No result | No | No result | No result | Cluster A4075149 mast adenovirus uncharacterized early protein | Zinc or mRNA binding | Auxiliary transport proteins |
| 16 | Q2KS67 | No result | No | No result | No result | Cluster A350866 early protein of human adenovirus B | 1. All DNA binding  2. Hydrolase | 1.Outer membrane |
| 17 | Q5EY73 | No result | No | No result | No result | Cluster A295505 human adenovirus B | 1. Hydrolases acting on ester bond  2. Lyases | ATP binding cassette (ABC) family |
| 18 | Q2KS66 | No result | No | No result | No result | Cluster A3609999  mast adenovirus early protein | 1. Metal binding  2. hydrolases act on peptide bonds  3. Lyases  4. Isomerases | Not found |
| 19 | I1V173 | No result | No | No result | No result | Cluster A3945367 early protein | 1. Zinc binding  2. All lipid binding protein | Plant defense |
| 20 | Q2KS62 | No result | No | No result | No result | Cluster mast adenovirus A2828072 | 1. Hydrolase acting on ester bond  2. Metal binding | Outer membrane |
| 21 | Q1L4D7 | No result | No | Adeno_52K | Multiple roles in association between viral and empty capsid | Cluster A3087289 probable DNA binding protein | Structural protein | Not found |
| 22 | I6LEV1 | No result | No | Adeno_52K | Required for DNA packaging and stable association | Cluster A380896 DNA binding | 1.Metal binding  2. Transferase | Not found |
| 23 | E1ARQ3 | No result | No | No result | No result | Cluster A3539386 Mast adenovirus | 1. Metal binding  2. All DNA binding | DNA repair  DNA replication |
| 24 | A6MLW9 | No result | No | No result | Glycerol-3-phosphate acyl-transferase | Cluster A4013018 early protein  hemorrhagic enteritis virus and hypothetical protein | 1. Transferases  2. RNA binding protein  3. Metal binding | 1.infflamatory response |
| 25 | A0A0B4SHT8 | No result | No | No result | No result | Cluster A350633 early protein | 1. Zinc binding  2. Metal binding | Not found |
| 26 | A0A0B4SJJ5 | No result | No | No result | No result | Cluster A4050149 adenoviridae early protein | 1. Zinc binding  2. mRNA binding | Auxiliary transport protein |
| 27 | A0A0B4SI61 | No result | No | No result | No result | Cluster A350866 uncharacterized early protein P-11335066 | 1. DNA binding 2. Metal binding | Outer membrane |
| 28 | A0A0B4SHQ0 | No result | No | No result | No result | Cluster A527920 early protein | 1. mRNA  2. Zinc binding | Auxiliary transport protein |
| 29 | Q2KS78 | No result | No | No result | No result | Cluster A3609999 human adenovirus early protein | 1. Metal binding  2. Hydrolysis acting on acid anhydrides |  |
| 30 | Q2KSC0 | No result | No | No result | No result | Cluster A3945467 adenovirus early protein | 1. Zinc binding  2. type II secretory pathway  3. Metal binding | Photosystem 1 |
| 31 | A0A0B4SIA5 | No result | No | No result | No result | Cluster A281985 adenovirus early protein | 1. Zinc binding  2. All lipid binding protein | Photosystem 1 |
| 32 | A0A0B4SGV2 | No result | No | No result | No result | Cluster A4263259 uncharacterized early protein | 1. Hydrolases acting on ester bond  2. Lyases | DNA repair |
| 33 | A0A0B4SIU9 | No result | No | No result | No result | Cluster A4263259 uncharacterized early protein | 1. Hydrolase acting on ester bond  2. Lyases C_C | 1.DNA repair |
| 34 | A0A0B4SH32 | No result | No | No result | Glycerol-3-phosphate acyl -transferase | Cluster A4013018 adenoviridae early protein | 1. Zinc Binding metal  2. All lipid binding protein | 1.plant defends |
| 35 | Q3ZKV3 | No result | No | No result | No result | Cluster A350633 human adenovirus B early protein | 1. Zinc binding  binding | Not found |
| 36 | Q3ZKV7 | No result | No | No result | No result | Cluster A350866 early protein | 1. All DNA binding  2. Metal binding | Outer membrane  P=58.6 |
| 37 | Q3ZKV4 | No result | No | No result | No result | Cluster A295505 early protein | 1. Hydrolases acting on ester bond  2. Lyases C-C | ATP binding Cassette (ABC) family |
| 38 | Q3ZKV2 | No result | No | No result | No result | Cluster A3945467 early protein | 1. Zinc binding  2. Metal binding | Not found |
