# Supplementary material for: Structural and functional annotation of hypothetical proteins of human adenovirus: prioritizing the novel drug targets
Source: BMC Res Notes. 2017 Dec 6;10:706. doi: 10.1186/s13104-017-2992-z (PMC5719520; doi:10.1186/s13104-017-2992-z)
Supplement: Supplementary file 6 — Additional file 6: Table S6. This table presents ligand binding sites prediction of hypothetical proteins of human adenovirus. [file 13104_2017_2992_MOESM6_ESM.docx]

| **Table S6: Ligand Binding Sites Prediction of Hypothetical Proteins of Human Adenovirus** | | | |
| --- | --- | --- | --- |
| **Sr No** | **Uniprot ID** | **C-score** | **Ligand Binding Sites** |
| 01 | P03269 | 0.6 | 10,11,12,13,60,62, 64,65,66,86, 89, 93, 96, 97, 149, 152, 546,550 |
| 02 | P03261 | 0.07 | 591,592 |
| 03 | P03263 | 0.09 | 34,35,38,75,79,82,83,86,93 |
| 04 | Q83127 | 0.12 | 42, 87, 88, 92, 93, 94 |
| 05 | Q1L4D7 | 0.07 | 18, 21 |
| 06 | I6LEV1 | 0.10 | 38, 43, 44, 107 |
