# Supplementary material for: Structural and functional annotation of hypothetical proteins of human adenovirus: prioritizing the novel drug targets
Source: BMC Res Notes. 2017 Dec 6;10:706. doi: 10.1186/s13104-017-2992-z (PMC5719520; doi:10.1186/s13104-017-2992-z)
Supplement: Supplementary file 7 — Additional file 7: Table S7. Sequence and structure motif prediction by pro-func. [file 13104_2017_2992_MOESM7_ESM.docx]

| **Table S7: Sequence and Structure Motif Prediction by Pro-Func** | | | |
| --- | --- | --- | --- |
| **Sr No** | **UniProt ID** | **Structure Motif** | **Sequence Motif** |
| 01 | P03269 | Ala159- Glu160- Arg161  Gly558-Arg559-Gly560  Leu406-Met407-Glu408  Gln241-Gln242-Ala243  Pro275-Asp276-Arg277 | Adenoviral DNA terminal protein |
| 02 | P03261 | Leu323-Gly324-Trp325-Asp326  His955-Thr956-Leu957  Ser926-Pro927-Pro928  Leu645-Gly646-Pro647  Lys850-Leu851-Asp852-Asn853 | DNA polymerase family B signature  DNA polymerase type B, organellar and viral  DNA-directed DNA-polymerase family B signature |
| 03 | P03263 | Ala105-Arg106-Ala107  Glu7-Leu8-Asp9  Asp4-Arg5-Glu6 | Adenoviral protein L1 52/55-kDa |
| 04 | Q83127 | Gln171-Asp172-Pro173 | Adenovirus E3 region protein CR2  Adenovirus E3 region protein CR1 |
| 05 | Q1L4D7 | Glu65-Ser66-Ala67  Val115Asn116-Gly117 | Adenoviral protein L1 52/55-kDa |
| 06 | I6LEV1 | Leu22-Tyr23-Leu24  Val98-Arg99-Glu100  Arg126-Tyr127-His128 | Adenoviral protein L1 52/55-kDa |
