# Supplementary figures and images for: Structural and functional annotation of hypothetical proteins of human adenovirus: prioritizing the novel drug targets
Source: BMC Res Notes. 2017 Dec 6;10:706. doi: 10.1186/s13104-017-2992-z (PMC5719520; doi:10.1186/s13104-017-2992-z)

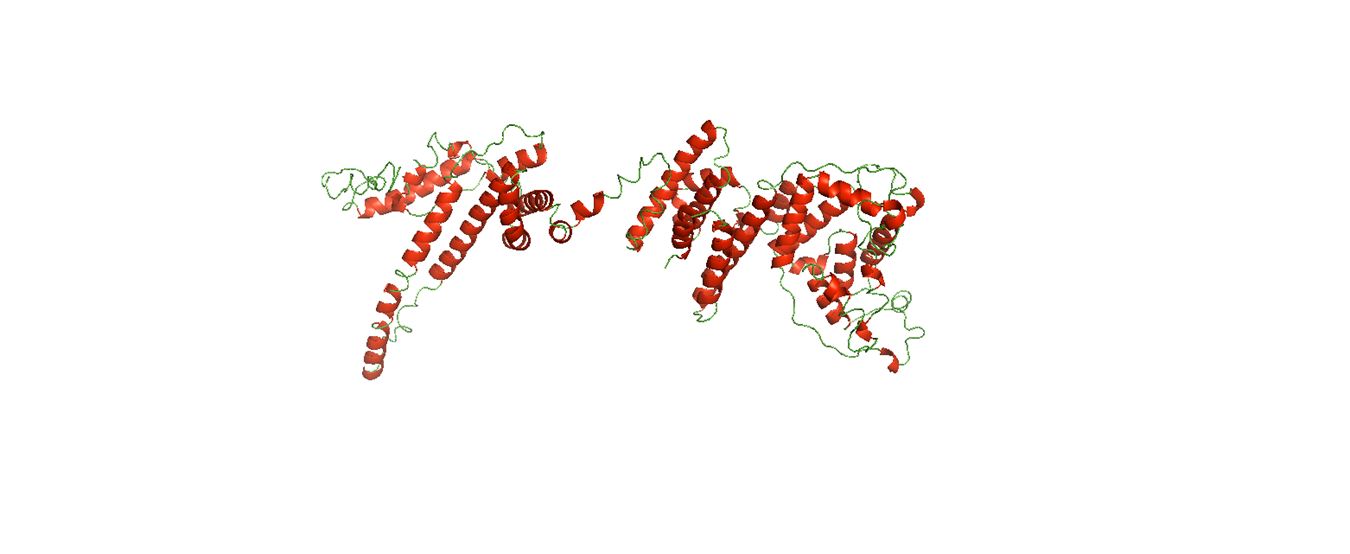

Supplement: Supplementary file 8 — Additional file 8: Figure S1. 3D structure of hypothetical protein P03269 predicted from I-TASSER. [file 13104_2017_2992_MOESM8_ESM.tif]

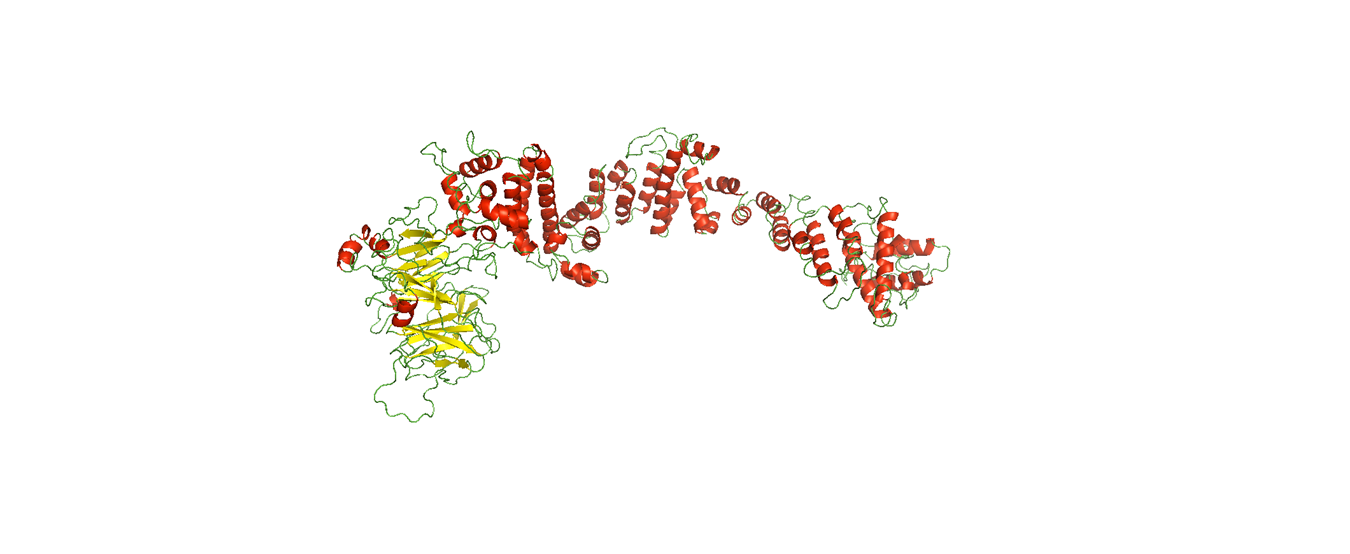

Supplement: Supplementary file 9 — Additional file 9: Figure S2. Evaluation of 3D structure of hypothetical protein P03269 through Ramachandran Plot. [file 13104_2017_2992_MOESM9_ESM.tif]

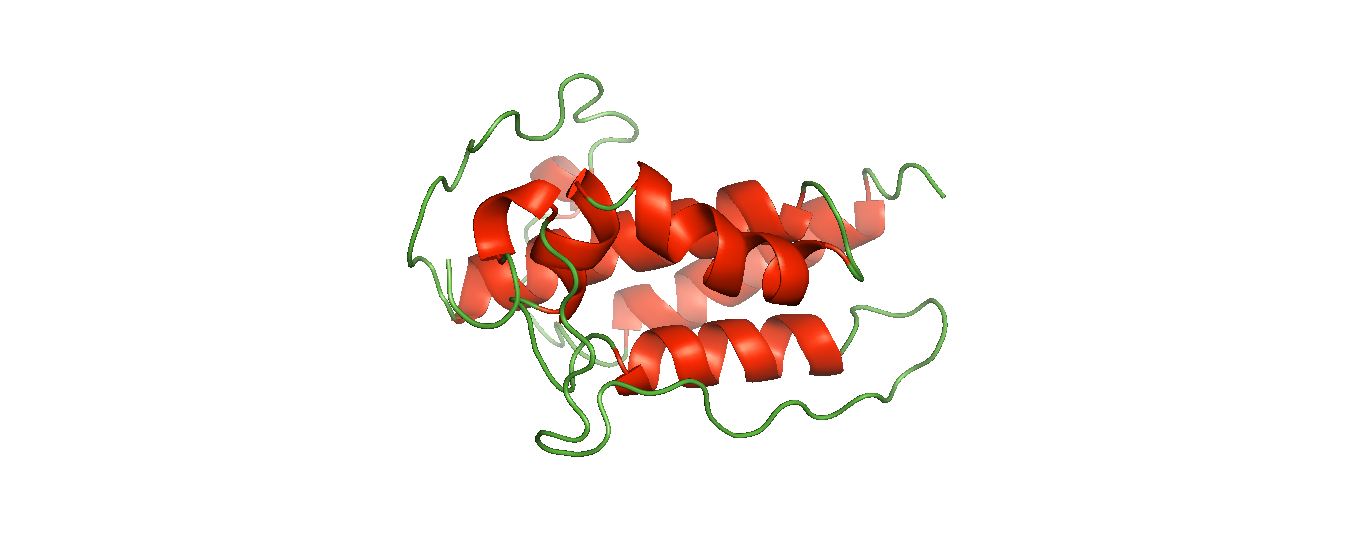

Supplement: Supplementary file 10 — Additional file 10: Figure S3. 3D structure of hypothetical protein P03261 predicted from I-TASSER. [file 13104_2017_2992_MOESM10_ESM.tif]

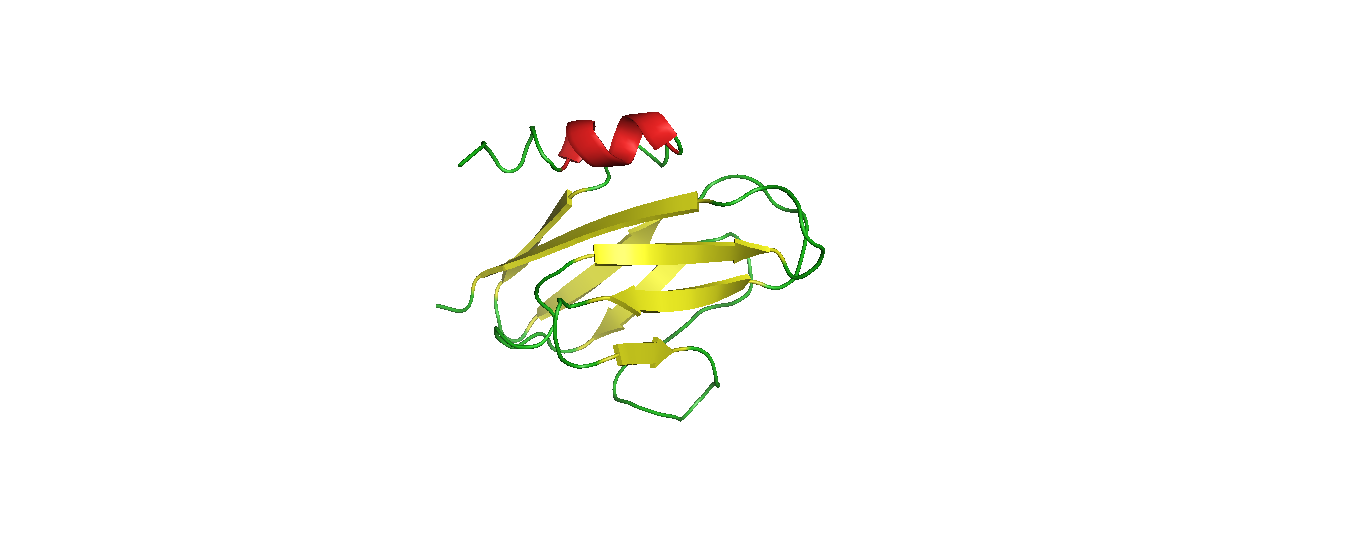

Supplement: Supplementary file 11 — Additional file 11: Figure S4. Evaluation of 3D structure of Hypothetical Protein P03261 through Ramachandran Plot [file 13104_2017_2992_MOESM11_ESM.tif]

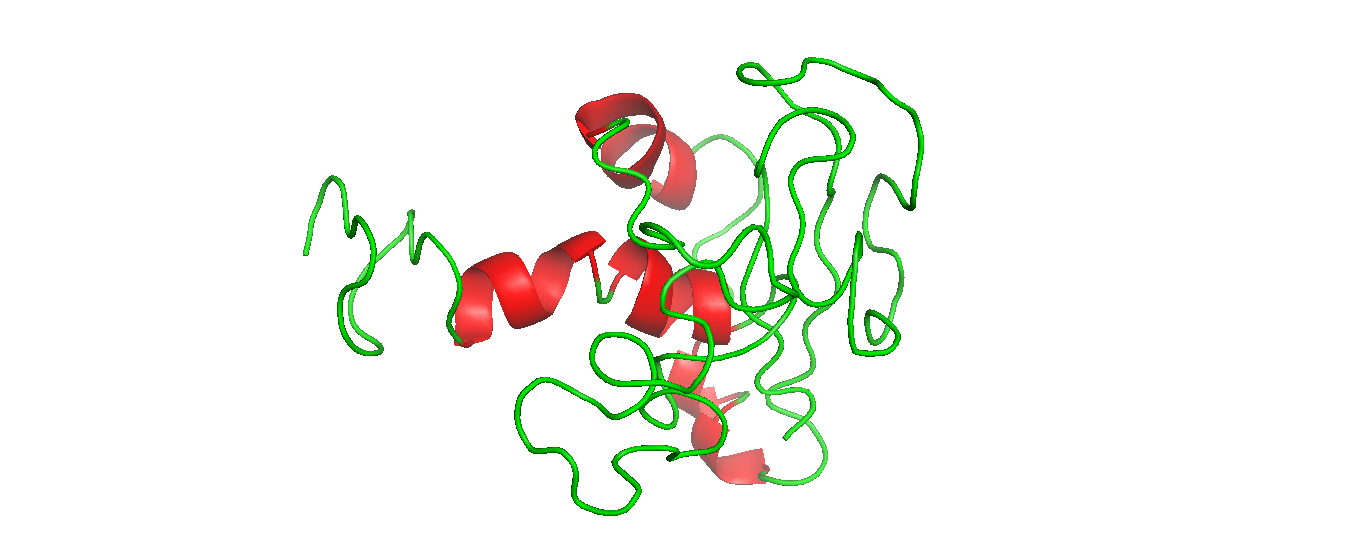

Supplement: Supplementary file 12 — Additional file 12: Figure S5. 3D structure of hypothetical protein P03263 predicted from I-TASSER. [file 13104_2017_2992_MOESM12_ESM.tif]

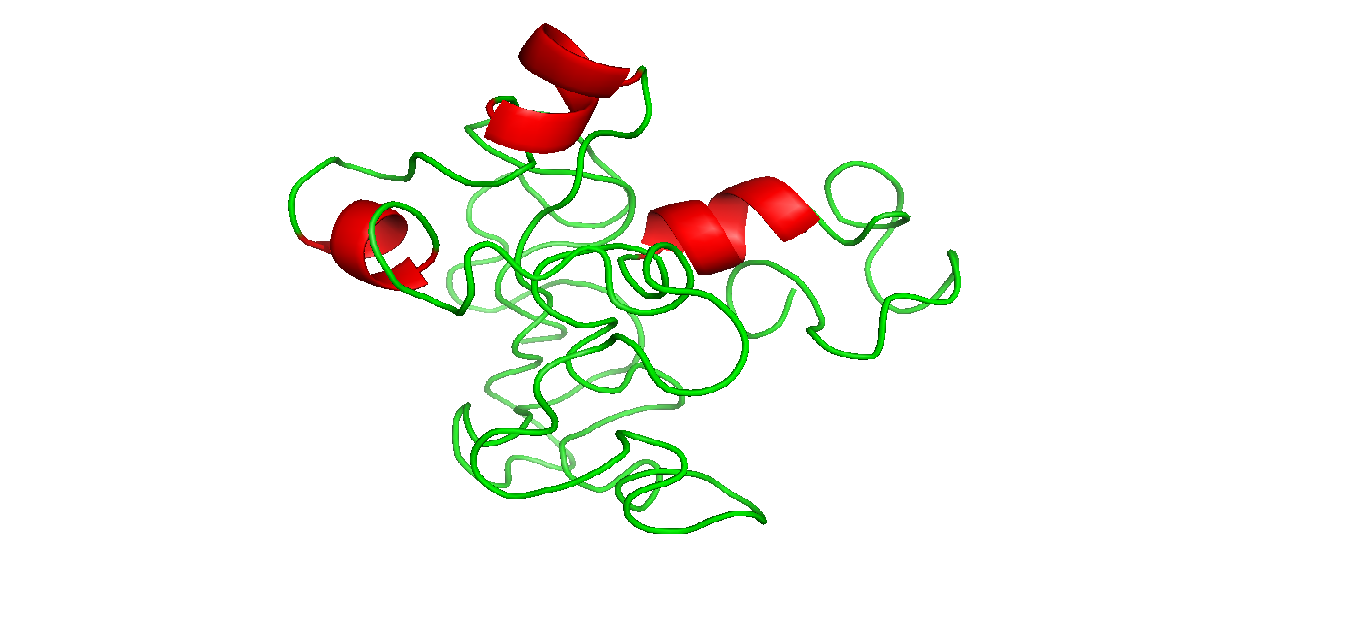

Supplement: Supplementary file 13 — Additional file 13: Figure S6. Evaluation of 3D structure of hypothetical protein P03263 through Ramachandran Plot. [file 13104_2017_2992_MOESM13_ESM.tif]

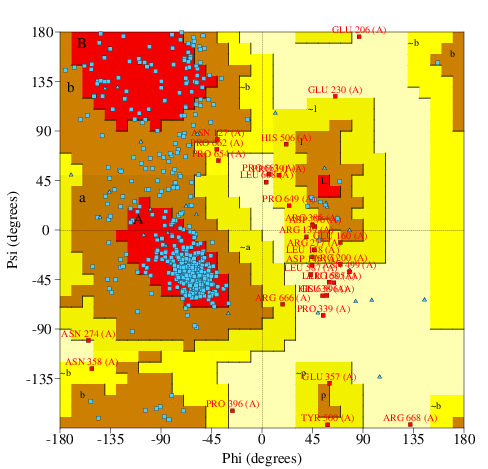

Supplement: Supplementary file 14 — Additional file 14: Figure S7. 3D structure of hypothetical protein Q83127 predicted from I-TASSER. [file 13104_2017_2992_MOESM14_ESM.tif]

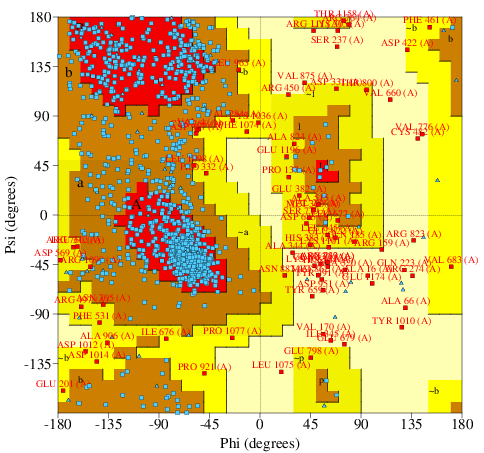

Supplement: Supplementary file 15 — Additional file 15: Figure S8. Evaluation of 3D structure of hypothetical protein Q83127 through Ramachandran Plot. [file 13104_2017_2992_MOESM15_ESM.tif]

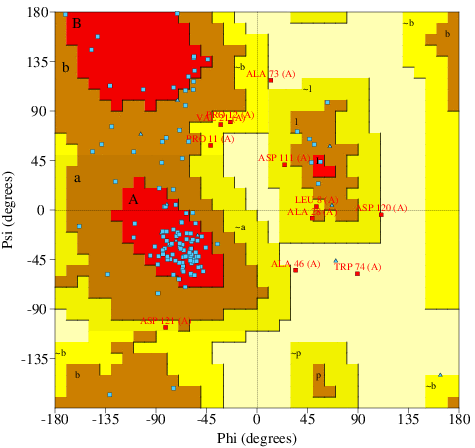

Supplement: Supplementary file 16 — Additional file 16: Figure S9. 3D structure of hypothetical protein Q1L4D7 predicted from I-TASSER. [file 13104_2017_2992_MOESM16_ESM.tif]

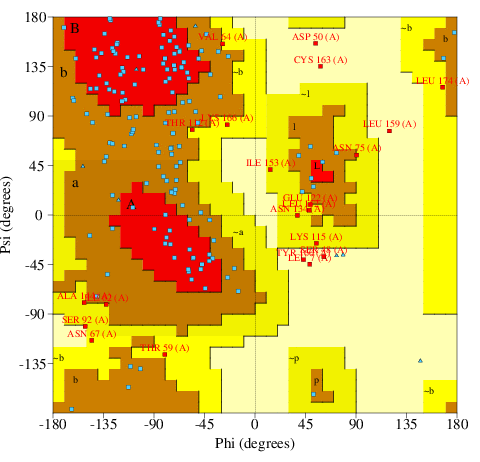

Supplement: Supplementary file 17 — Additional file 17: Figure S10. Evaluation of 3D structure of hypothetical protein Q1L4D7 through Ramachandran Plot. [file 13104_2017_2992_MOESM17_ESM.tif]

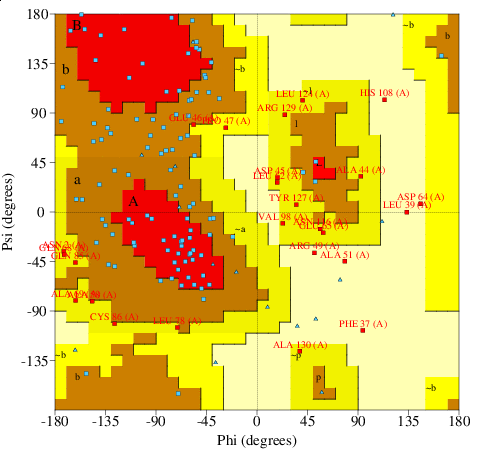

Supplement: Supplementary file 18 — Additional file 18: Figure S11. 3D structure of hypothetical protein 16LEV1 predicted from I-TASSER. [file 13104_2017_2992_MOESM18_ESM.tif]

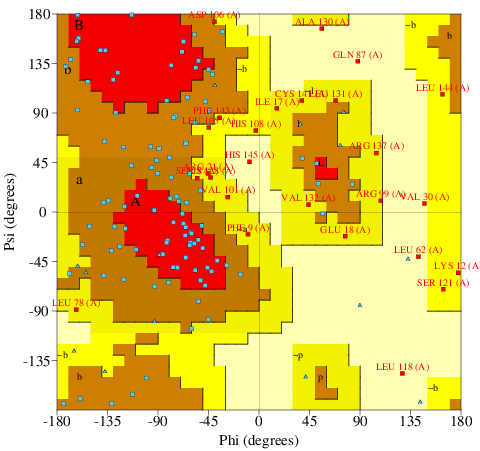

Supplement: Supplementary file 19 — Additional file 19: Figure S12. Evaluation of 3D structure of hypothetical protein I6LEV1 through Ramachandran Plot. [file 13104_2017_2992_MOESM19_ESM.tif]
